# Pregnancy- and Lactation-Associated Osteoporosis: A Literature Review Based on a Clinical Case

**DOI:** 10.7759/cureus.72889

**Published:** 2024-11-02

**Authors:** Filipa Abelha Pereira, Diana Oliveira Miranda, José Miguel Alvarenga, Ana Lucinda Correia

**Affiliations:** 1 Internal Medicine, Santo António University Hospital Center, Porto, PRT; 2 Dermatology, Santo António University Hospital Center, Porto, PRT; 3 Orthopedics and Traumatology, Santo António University Hospital Center, Porto, PRT

**Keywords:** lactation, osteoporosis, osteoporotic fractures, pregnancy, pregnancy complications

## Abstract

Pregnancy- and lactation-associated osteoporosis (PLO) is a rare condition that affects women during pregnancy or postpartum. We report the case of a 29-year-old woman who experienced severe lower back pain 11 weeks after childbirth, revealing multiple compressive vertebral fractures in imaging studies. Despite no identifiable risk factors, the patient underwent an assessment ruling out secondary causes of osteoporosis, with only vitamin D deficiency and expected alterations in bone biomarkers identified. Treatment involved chemically suppressing lactation, along with calcium and vitamin D supplementation. After three months of follow-up, vitamin D levels and bone biomarkers normalized, but the pain persisted, and bone mineral density decreased. The literature review underscores the lack of understanding regarding PLO's etiology and pathophysiology. Possible causes include genetic factors, alterations in calcium metabolism, and abnormalities in calcium transfer adaptation from the mother to the fetus. Treatment lacks standardization, but calcium and vitamin D supplementation are common. Therapies such as bisphosphonates, teriparatide, calcitonin, denosumab, and strontium ranelate have been evaluated, considering their effectiveness and safety. Due to the initial lack of improvement, teriparatide was initiated in this case. In conclusion, PLO poses a diagnostic challenge due to its low incidence and lack of comprehension of underlying mechanisms. Early diagnosis, personalized treatment, and strict monitoring are crucial for improving the prognosis of these patients.

## Introduction

Osteoporosis is a systemic skeletal disease characterized by a decrease in bone mineral density (BMD) and deterioration of bone tissue microarchitecture, resulting in increased bone fragility and susceptibility to pathological fractures [1]. BMD refers to the amount of mineral matter per square centimeter of bones and is a key indicator of bone strength and health. However, BMD alone does not fully capture bone quality; therefore, additional assessment tools like the Trabecular Bone Score (TBS) are used. TBS is a gray-level textural measurement derived from dual-energy X-ray absorptiometry (DEXA) images that evaluates bone microarchitecture, particularly the trabecular structure. It provides information about bone quality independent of BMD, with lower TBS values indicating degraded microarchitecture and higher fracture risk [1].

Pregnancy- and lactation-associated osteoporosis (PLO) is a particularly rare form of osteoporosis that typically occurs during the third trimester of pregnancy or the postpartum period [2]. It was first described by Nordin and Roper in 1955 [3]. The incidence of PLO is estimated to be approximately 0.4 per 100,000 women, although a significant number of cases likely remain undiagnosed due to its subtle presentation and overlap with common postpartum conditions [4].

During pregnancy and lactation, significant physiological changes occur to meet the calcium demands of the developing fetus and breast milk production. Typically, the body adapts by increasing intestinal calcium absorption and, to a lesser extent, by mobilizing calcium from the maternal skeleton, which can lead to temporary decreases in BMD. These changes are usually reversible after weaning, and most women do not experience long-term bone health issues. However, in rare cases, these adaptations may not suffice or may be exacerbated by other factors, leading to substantial bone loss and the development of PLO.

The main symptom of PLO is severe prolonged lower back pain, and the most serious complication is fragility fractures, often located in the spine [2]. The etiology and pathophysiology of PLO are not yet fully understood, and there is no standard treatment. Several factors have been proposed as possible causes, including genetic factors, changes in calcium metabolism, and abnormalities in the adaptation of calcium transfer from the mother to the fetus. Additionally, treatment can vary widely, with options including calcium and vitamin D supplementation and the use of medications such as bisphosphonates, teriparatide, calcitonin, denosumab, and strontium ranelate.

In this article, we present a literature review on PLO in the context of a severe clinical case involving a 29-year-old woman, with no relevant personal history, who developed multiple compressive vertebral fractures 11 weeks postpartum. 

This bibliographic review is based on research conducted in various databases, such as PubMed, Scopus, and Cochrane, using Medical Subject Headings (MeSH) terms and keywords related to osteoporosis, pregnancy, and lactation. We included case reports, case series, and studies on changes in bone metabolism during pregnancy and lactation, with a preference for articles published in the last 10 years but also incorporating relevant older studies. Our aim is to provide a comprehensive overview of the epidemiology, diagnosis, risk factors, treatment strategies, and possible recurrences of PLO, highlighting the need for early diagnosis, individualized treatment, and rigorous follow-up to improve the prognosis of these patients.

The objective of this literature review and case study is to provide a comprehensive overview of PLO, highlighting key aspects of its epidemiology, diagnosis, risk factors, treatment strategies, and potential recurrences. Additionally, we aim to report a severe case of PLO to illustrate the challenges associated with its diagnosis and management. It is important to note the limitations of this review, such as the reliance on case reports and the limited number of available studies, which may impact the generalizability of the findings.

## Case presentation

We present the case of a 29-year-old woman, previously healthy, primigravida, who had an uncomplicated full-term delivery and was breastfeeding. Eleven weeks postpartum, she was admitted to the emergency department due to severe lower back pain that had progressively worsened over four to six weeks, impacting her daily functioning and sleep quality, and was refractory to non-steroidal anti-inflammatory drugs. There was no history of trauma. On admission, no changes were noted in the physical examination, except for pain on palpation of the dorsal spine in the lumbosacral region. An X-ray detected flattening of L11 and L12, and a computed tomography documented decreased bone texture and a reduction in the height of 10 vertebral bodies, both dorsal and lumbar, due to depression of the platforms (D5, D6, D7, D8, D10, D12, L1, L2, L3, and L5). She was evaluated by Orthopedics, who recommended a Jewett brace for comfort.

Hospitalization for etiological study and pain control was proposed. DEXA confirmed decreased BMD, particularly in the lumbar spine, with a TBS of 1.171, which is suggestive of degraded microarchitecture compared to the reference population. As shown in Table 1, BMD values at diagnosis were significantly below the expected range for age, with a Z-score of -4.5 in the lumbar spine. Follow-up at three months showed only a minimal improvement in lumbar spine BMD (0.559 g/cm² from 0.554 g/cm²) and a decline in BMD at the femoral neck and total proximal femur, highlighting the progressive nature of the condition despite initial treatment.

**Table 1:** DEXA at the time of diagnosis and reassessment at three months of follow-up DEXA: dual-energy X-ray absorptiometry; BMD: bone mineral density; SD: standard deviation Z-score is a comparison of the patient's BMD to an age-matched population. Values <-2 SD are considered below the expected range for age

	Lumbar spine		Femoral neck		Total proximal femur	
	Diagnosis	Three months	Diagnosis	Three months	Diagnosis	Three months
BMD (g/cm^2^)	0.554	0.559	0.595	0.538	0.747	0.705
Reference values (BMD)	1.1-1.2		0.9-1.0		0.9-1.1	
Z-score (SD)	-4.5	-4.4	-2.2	-2.7	-1.6	-1.9
Reference values (Z-score)	Between -2.0 and +2.0					

Personal history of fractures and family history of osteoporosis or osteoporotic fractures were excluded. The patient is of normal weight (BMI 20.9 kg/m²), with no prior clinical signs suggestive of vitamin deficiencies or malabsorption syndrome. The patient only reported intolerance to milk protein. Regarding the pregnancy, there were no complications or periods of prolonged immobilization, and she received adequate supplementation with folic acid, iodine, and iron. No other medications were taken during pregnancy. Secondary causes of osteoporosis such as hypercortisolism, hyperthyroidism (thyroid-stimulating hormone (TSH), triiodothyronine (T3L), and thyroxine (T4L) levels were normal), hyperparathyroidism (parathyroid hormone (PTH) level was normal), celiac disease, or autoimmune conditions, neoplastic or lymphoproliferative diseases, as well as bone tuberculosis were excluded. No vitamin deficiencies were identified except for a vitamin D deficiency (25.50 nmol/l) (Table 2). Serum calcium, corrected for albumin, was within the normal range (2.29 mmol/l), and urinary calcium excretion was also normal (4.79 mmol/24 h) (Table 2). Serum phosphorus was marginally above the upper limit (1.47 mmol/l) but normalized during hospitalization. These laboratory findings helped rule out secondary causes and supported the diagnosis of PLO.

**Table 2:** Laboratory measurements of phosphocalcic metabolism indicators and exclusion of secondary causes of osteoporosis at the time of diagnosis PTH: parathyroid hormone; TSH: thyroid-stimulating hormone; T3L: triiodothyronine; T4L: thyroxine

	Reference values	Values at the time of diagnosis
Serum calcium, corrected for albumin (mmol/l)	2.15-2.50	2.29
Serum phosphorus (mmol/l)	0.87-1.45	1.47
Vitamin D (25-OH) (nmol/l)	50-150	25.50
PTH (pg/ml)	15-65	18.8
TSH (µUI/ml)	0.30-3.94	0.85
T3L (pg/ml)	2.42-4.36	3.46
T4L (ng/dl)	0.95-1.57	1.30
Cortisol (ug/dl)	6.2-19.4	17.8
Urinary calcium (mmol/24 h)	2.50-8.00	4.79

Biochemical markers of bone turnover, products released by osteoblasts and osteoclasts, and collagen type 1 degradation precursors and products were measured. Bone formation markers included bone-specific alkaline phosphatase, osteocalcin, and pro-collagen peptides. Resorption markers included tartrate-resistant acid phosphatase and collagen degradation products. At the time of diagnosis, there was a significant elevation in bone resorption markers, specifically beta CTX (C-terminal cross-links of type 1 collagen; 0.792 ng/ml) and tartrate-resistant acid phosphatase (1035 nmol/h/ml), consistent with the increased bone resorption associated with lactation (Table 3). Bone formation markers, such as osteocalcin (29.78 ng/ml) and P1NP (67.27 µg/l), were also within expected levels during lactation, indicating active bone turnover (Table 3).

**Table 3:** Bone turnover markers at the time of diagnosis and at three months of follow-up *: reference values in pre-menopause; Beta CTX: C-terminal cross-links of type 1 collagen; P1NP: N-terminal propeptide of type 1 procollagen

	Reference values	Values at the time of diagnosis	Values after three months
Bone formation markers			
Osteocalcin (ng/ml)	11.0-46.0	29.78	36.89
Bone alkaline phosphatase (µg/l)	<22.4	17.9	-
P1NP (ug/l)	16.3-73.9	67.27	80.46
Bone resorption markers			
Beta CTX (ng/ml)	0.025-0.573*	0.792	0.488
Tartrate-resistant acid phosphatase (nmol/h/ml)	54-815	1035	453

At three months of follow-up, normalization of vitamin D levels (129.00 nmol/l) and bone turnover biomarkers was observed (Table 3 and Table 4). Specifically, beta CTX levels decreased to 0.488 ng/ml, and tartrate-resistant acid phosphatase levels reduced to 453 nmol/h/ml, indicating a reduction in bone resorption activity (Table 3). Serum phosphorus also returned to normal levels (1.12 mmol/l), and PTH levels remained within the normal range, which further confirmed the normalization of bone metabolism (Table 4).

**Table 4:** Laboratory measurements of phosphocalcic metabolism indicators and exclusion of secondary causes of osteoporosis at three months of follow-up Free T3: triiodothyronine; Free T4: thyroxine; PTH: parathyroid hormone; TSH: thyroid-stimulating hormone

	Reference values	Values at the time of diagnosis	Values after three months
Serum calcium, corrected for albumin (mmol/l)	2.15-2.50	2.29	2.27
Serum phosphorus (mmol/l)	0.87-1.45	1.47	1.12
Vitamin D (25-OH) (nmol/l)	50-150	25.50	129.00
PTH (pg/ml)	15-65	18.8	36.6
TSH (µUI/ml)	0.30-3.94	0.85	0.65
Free T3 (pg/ml)	2.42-4.36	3.46	3.43
Free T4 (ng/dl)	0.95-1.57	1.30	1.08

However, the patient continued to experience pain and functional limitations, and the DEXA scan showed a worsening of -5.6% in the total proximal femur and no significant changes in the lumbar spine, as shown previously in Table 1. Teriparatide therapy was initiated in follow-up by the Endocrinology department.

## Discussion

This report presents a severe case of PLO with multiple compressive vertebral fractures. The patient presented with severe and progressive lower back pain and functional limitation. The diagnosis was made in an emergency setting, at the first contact with our institution, following an evaluation of the complaints and a computed tomography scan. She had previously been assessed in another context, without available imaging, and without suspicion of the diagnosis. Due to the very low incidence, the diagnosis may be delayed in the absence of clinical suspicion. However, given the potential severity, it is important for healthcare professionals to keep this condition in mind when encountering young women who are pregnant or in the postpartum period with severe lower back pain. Although back pain is common in the late stages of pregnancy, pain associated with the presence of vertebral body fractures is significantly debilitating. It is crucial to recognize specific red-flag symptoms that warrant further investigation for PLO, including non-response to non-steroidal anti-inflammatory drugs: persistent back pain unresponsive to conventional analgesics should raise suspicion for underlying conditions like PLO; and functional limitations, significant impairments in daily activities, particularly when accompanied by severe pain, are critical indicators for considering more serious diagnoses. In the case of prepartum women, the use of magnetic resonance imaging may facilitate diagnosis [5].

Several factors have been identified as risks for the development of PLO, including family history (osteoporosis and/or osteoporotic fractures in grandparents, mother, or sisters); menstrual irregularities; low body weight, restrictive diets, or anorexia; smoking and alcohol use; certain comorbidities such as connective tissue or bone diseases and inflammatory bowel disease; and specific medications such as corticosteroids or heparins [2]. Some factors also considered predisposing during pregnancy include prolonged bed rest and uncontrollable vomiting during the first trimester [5]. Most reports and case series fail to identify the cause of PLO [6,7]. However, a retrospective study of 52 women in France identified risk factors for low BMD prior to or during pregnancy in 67% of the participants [5]. No risk factors were identified in the case presented.

When no cause or predisposing factor can be identified, it may be hypothesized that there was a low BMD prior to pregnancy, where a normal calcium metabolic demand could lead to disease, or that there are abnormalities in the adaptive process of transferring calcium from the mother to the fetus [5]. Fetal skeletal formation requires placental calcium transfer, with 80% of fetal calcium needs occurring in the third trimester. If the maternal skeleton were the only source of calcium, the risk of bone mass loss would be very high; thus, adaptive mechanisms to meet metabolic demands are in place [5]. Lactation also represents an additional metabolic demand [8].

The pathophysiological mechanisms associated with PLO are not fully understood. Several hypotheses have been proposed, including alterations in calcium metabolism, hereditary factors, and genetic mutations [2,8,9].

Family studies suggest the presence of hereditary factors [2]. Peris et al. [10], in an investigation of first-degree relatives of women with PLO, suggested a strong genetic component: 53% of the relatives of PLO patients had osteoporosis, a statistically significant difference (p<0.05) compared to 15% of controls. In a multicenter study in Germany [11], a cohort of 42 women with PLO was screened for a genetic panel including genes relevant to skeletal disorders. Heterozygous variants classified as disease-causing were found in 19% of the women, and heterozygous variants of interest were found in 50%. The disease-causing variants were identified in the genes LRP5, WNT1, ALPL, COL1A1, COL1A2, and SLC34A3. Several variants, particularly in the genes LRP5 and WNT1, are associated with a state of low bone tissue remodeling. Mutations in LRP5 and WNT1 impair bone formation signaling pathways, leading to reduced bone mass and increased fragility [11]. Additionally, variants in the COL1A1 and COL1A2 genes, which encode type I collagen, contribute to compromised bone quality and increased susceptibility to fractures [11]. ALPL mutations impair bone mineralization by affecting alkaline phosphatase activity, further contributing to the pathogenesis of PLO [11]. Cohen et al. [12] examined the rate of bone remodeling based on iliac crest biopsies and showed that women with PLO had a significantly lower remodeling rate compared to premenopausal women, with the assessment done at least one year after delivery, which would be representative of their baseline state. These results seem to suggest that abnormal osteoblast function or other defects in the bone remodeling process may contribute to PLO. Additionally, genetic mutations affecting bone signaling and remodeling pathways, combined with the hereditary predisposition observed in family studies, suggest a multifactorial etiology involving both genetic and environmental factors. Genetic counseling and screening may be beneficial in families with a history of PLO or related conditions, particularly for genes such as LRP5, WNT1, and COL1A1, which have a known impact on bone metabolism [11].

Alterations in calcium metabolism during pregnancy may contribute to PLO. About six weeks before delivery, fetal needs are around 300-500 mg of calcium daily, in addition to the needs of the pregnant woman herself [13]. During pregnancy, to maintain a positive balance, there is a doubling of intestinal calcium absorption [14]. Insufficient calcium intake and the development of secondary hyperparathyroidism increase bone resorption. Clinical evidence indicates that parathyroid hormone-related protein (PTHrP) plays a significant role in bone resorption during pregnancy, being excreted in large quantities by mammary and placental tissues during the third trimester [15]. In a normal pregnancy, increased production of PTHrP can lead to increased bone resorption, with its role being amplified in situations of low calcium intake. In the presented case, a thorough assessment of the patient's habitual diet, both before and during pregnancy, was carried out and was interpreted as balanced and capable of providing an adequate amount of calcium.

Lactation also represents a calcium metabolic demand; however, intestinal calcium absorption progressively decreases to baseline levels after delivery [13,14]. The calcium in breast milk comes from bone resorption. This process involves prolactin, whose production increases in the postpartum period, and the reflex arcs triggered by sucking during breastfeeding. Both prolactin and these reflex arcs lead to the inhibition of the hypothalamic-pituitary-gonadal axis, resulting in the reduced production of gonadotropins and ovarian function, and a decrease in estradiol production. This decrease in estradiol levels, in turn, stimulates the production of osteoclasts and enhances their function. Additionally, the combination of these changes (increased prolactin, sucking reflex arcs, decreased estradiol) leads to the production of PTHrP by the mammary tissue [16].

The treatment of PLO is not standardized, likely due to its low incidence and the lack of understanding of the pathophysiological mechanisms. Deciding on the appropriate therapeutic strategy can be challenging. In the reported case, lactation was stopped, a balanced diet and sun exposure were recommended, and supplementation with calcium and vitamin D was initiated. There is consensus on the importance of a balanced diet and, for women undergoing breastfeeding, the potential importance of discontinuing it, as it corresponds to increased bone resorption [17,18].

Supplementation with vitamin D and calcium seems vital for maintaining bone tissue homeostasis. Adequate levels of vitamin D require proper sun exposure or supplementation with 600-1000 IU of vitamin D3. Supplementation in individuals with osteoporosis should include calcium (1200 mg/day) and vitamin D.

Antiresorptive therapy with bisphosphonates prevents bone loss in young women and is well tolerated and effective in cases of PLO appearing to confer greater increases in BMD than those observed in patients treated with supplementation alone [19]. However, data on their safety and long-term fracture risk reduction in premenopausal women are scarce. Bisphosphonates have a long half-life and accumulate in the skeleton, raising concerns about their use in women wishing to conceive due to potential risks to the fetus. Animal studies have shown toxic effects in pregnant mice due to accumulation in both the mother's and fetus' skeletons [2]. Clinical data in humans are limited. Sokal et al. [20] found no evidence of major teratogenic effects in 36 women exposed to bisphosphonates during pregnancy. However, they reported an increase in neonatal complications in women with systemic diseases and an increase in spontaneous abortions in women with skeletal diseases. Green and Pappas [21], in a review of case reports and other published data on fetal and neonatal effects associated with maternal bisphosphonate use, found no evidence of severe adverse fetal or neonatal effects, though they noted the limited amount of published data on the subject. The British Society for Rheumatology considers that there is insufficient data to recommend a specific timing for stopping bisphosphonate before conception but recommends discontinuation three months prior to pregnancy [22]. In our patient's case, given her desire for future pregnancies and after multidisciplinary discussion, the decision was made not to initiate bisphosphonate therapy due to the potential risks to the fetus and the lack of sufficient safety data in premenopausal women.

Teriparatide, a recombinant form of human parathyroid hormone, acts as an osteoanabolic agent, increasing BMD and reducing the incidence of fragility fractures in postmenopausal women and cases of glucocorticoid-induced osteoporosis. It has also been evaluated in premenopausal women with osteoporosis, where increases in lumbar spine BMD were significantly greater compared to treatment with alendronate [23]. Several reports describe women with PLO treated with teriparatide showing significant improvement in pain and an increase in BMD [24-26]. Unlike bisphosphonates, teriparatide does not accumulate in the skeleton and has a short half-life, making it less likely to affect a fetus conceived after its discontinuation [27]. However, teriparatide is not indicated during pregnancy due to unknown effects on fetal development, and it is not known whether it is excreted in breast milk [2]. In our patient's case, teriparatide was started at three months of follow-up due to the absence of clinical improvement with conservative management. Given her desire for future pregnancies, teriparatide was chosen over bisphosphonates because it does not accumulate in bone tissue, potentially reducing fetal exposure after discontinuation. The decision was made after a thorough risk-benefit analysis, considering the severity of her osteoporosis, the potential for significant improvement in BMD, and the need to minimize risks associated with long-term medication use. Close monitoring was planned during therapy to address any potential side effects and to ensure the safety and efficacy of the treatment.

Calcitonin is a hormone that inhibits bone resorption, and there are reports of its use in women with PLO [28]. However, the use of calcitonin is associated with a statistically significant increase in the risk of developing malignant neoplasms. The risk is associated with prolonged use (studies vary from six months to five years), so exposing a young patient to this risk should be carefully considered.

Denosumab is a human monoclonal antibody that inhibits the activation of osteoclasts and their precursors. This leads to the suppression of bone turnover, and its therapeutic effect in cases of postmenopausal osteoporosis is well documented. Its use in patients with PLO has also been reported, with significant improvement in pain and considerable increases in BMD [29,30]. A study with cynomolgus monkeys that received subcutaneous denosumab during organogenesis, at doses higher than the recommended human doses, showed no evidence of maternal toxicity or fetal harm [31]. Ijuin et al. [30] conclude that, in addition to weaning, the administration of teriparatide followed by denosumab led to a considerable improvement in symptoms and BMD, suggesting that this could be a promising choice for the treatment of PLO.

Strontium ranelate is approved for the prevention of fragility fractures in postmenopausal women and in cases of osteoporosis in men; however, it is reserved for severe cases where other therapeutic options are limited due to cardiovascular and cerebrovascular adverse effects. There is no data on the use of strontium ranelate in pregnant women. At high doses, animal studies have revealed reversible effects on the bone of offspring from mice and rabbits treated during gestation. Physicochemical data suggest that the drug is excreted in human breast milk. Tanriover et al. were the first to use strontium ranelate in the treatment of PLO and reported good pain control alongside a significant increase in BMD [2,32].

Vitamin K has also been used as a therapeutic option in cases of PLO. Several studies highlight the role of vitamin K in bone health and the prevention of osteoporotic fractures; however, the results are mostly inconclusive [33].

Regarding the possible surgical approach for compressive vertebral fractures, such as percutaneous kyphoplasty or vertebroplasty, due to the lack of knowledge about the potential long-term sequelae of the procedure itself, conservative management is currently considered the first-line approach. For hip fractures, surgical intervention with anatomical reconstruction is crucial in preventing long-term complications [2].

Some authors also suggest that, given the recovery of BMD after childbirth, an expectant and monitoring approach may be appropriate [2].

The recurrence of PLO in future pregnancies is a significant concern that patients should be informed about. Kyvernitakis et al. [6] reported that nearly 25% of patients with PLO experience a subsequent fracture within a six-year follow-up period. This fracture risk appears to be related to the number of fractures at the time of diagnosis.

There are potential long-term consequences, such as chronic pain and irreversible static postural changes, highlighting the need for the diagnosis, treatment, and ongoing monitoring of this condition.

This case illustrates the challenges of delayed diagnosis due to the rarity and atypical presentation of PLO, highlighting the need for greater clinical awareness. The progression of the disease, despite initial conservative treatment, underscores the importance of timely escalation to more aggressive interventions, such as teriparatide. The case emphasizes the value of personalized, closely monitored treatment plans and demonstrates the necessity of adapting strategies based on individual disease progression. These insights contribute to a better framework for managing similar cases, emphasizing early diagnosis, prompt supplementation, and consideration of anabolic agents when conventional therapy is insufficient.

Given the potential long-term consequences, such as chronic pain and irreversible postural changes, it is crucial not only to emphasize the need for the diagnosis, treatment, and ongoing monitoring of this condition but also to consider preventive measures. Preventive strategies may include ensuring adequate calcium and vitamin D intake, engaging in appropriate weight-bearing physical activities, and providing education on bone health to at-risk women during pregnancy and lactation. Early intervention and lifestyle modifications can help mitigate the progression of osteoporosis and reduce the risk of fractures.

## Conclusions

PLO is a rare form of osteoporosis that can lead to multiple osteoporotic fractures and is associated with the development of chronic pain. This case illustrates the challenges of delayed diagnosis due to the rarity and atypical presentation of PLO, highlighting the need for greater clinical awareness, along with an appreciation of potential warning signs. The progression of the disease, despite initial conservative treatment, underscores the importance of timely escalation to more aggressive interventions. Personalized and closely monitored treatment plans are crucial, demonstrating the necessity of adapting strategies based on individual disease progression. These insights contribute to a better framework for managing similar cases, emphasizing that early diagnosis, prompt supplementation, and consideration of anabolic agents when conventional therapy is insufficient can improve patient outcomes.
